# Ovarian characteristics and in vitro nuclear and cytoplasmic oocyte maturation in Duroc and Landrace pigs

**DOI:** 10.1002/vms3.498

**Published:** 2021-05-03

**Authors:** Reina Jochems, Ann Helen Gaustad, Louisa J. Zak, Eli Grindflek, Teklu T. Zeremichael, Irma C. Oskam, Frøydis D. Myromslien, Elisabeth Kommisrud, Anette K. Krogenæs

**Affiliations:** ^1^ Norsvin SA Hamar Norway; ^2^ Faculty of Veterinary Medicine Norwegian University of Life Sciences Oslo Norway; ^3^ Topigs Norsvin Research Center Beuningen The Netherlands; ^4^ Department of Biotechnology Inland Norway University of Applied Sciences Hamar Norway; ^5^ The Animal Production Experimental Centre Norwegian University of Life Sciences Ås Norway

**Keywords:** breed differences, Duroc, in vitro oocyte maturation, Landrace, ovary, porcine

## Abstract

Differences in total number of piglets born per litter are observed between the Norwegian Duroc (ND) sire and Norwegian Landrace (NL) dam line. The aim of this study was to evaluate ovarian characteristics, and in vitro nuclear and cytoplasmic oocyte maturation in both breeds. One day after weaning, follicular phase ovaries were collected. Ovary length and weight were measured and the number of follicles (< 3 mm and 3–8 mm) was counted. Cumulus‐oocyte complexes (COCs) were collected and matured for 48 hr. To assess cumulus expansion, COC area was analysed at 0 and 20 hr. Nuclear maturation and cortical granule (CG) distribution were analysed at 20 and 48 hr, and total glutathione (GSH) was measured at 48 hr to further elucidate cytoplasmic maturation. In first parity sows, a smaller ovary length and fewer 3 to 8 mm follicles were observed in ND compared to NL. For all sows, ND COCs covered a significantly smaller area at 0 hr, but a higher cumulus expansion ratio was observed at 20 hr compared to NL (364 ± 46% versus. 278 ± 27%, *p* < 0.001). At 20 hr, more ND oocytes exhibited advanced stages of nuclear maturation, while more NL oocytes showed advanced stages of CG distribution. Nuclear maturation to MII stage at 48 hr did not differ between ND and NL oocytes (90.1% and 87.7%, respectively). Moreover, no significant differences were observed for GSH content or CG distribution after maturation. In conclusion, differences with regard to ovarian characteristics as well as to cumulus expansion, and nuclear and cytoplasmic oocyte maturation at 20 hr were observed between the breeds. Further studies are required to determine if this subsequently affects in vitro fertilization and embryo development.

## Graphical abstract

Differences in reproduction traits are observed between the Norwegian Duroc sire line and Norwegian Landrace dam line, which could affect in vitro embryo production outcomes. In this study, differences with regard to ovarian characteristics as well as to cumulus expansion, and nuclear and cytoplasmic oocyte maturation at 20 h were observed between the two breeds. Further experiments are required to study if there are differences in in vitro fertilization and embryo development.

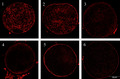

## 1 INTRODUCTION

In Norway, the farmer‐owned cooperative Norsvin has been in charge for all pig breeding since 1958. Since the beginning, a breeding program was established for the Norwegian Landrace (NL) dam line and it has therefore a long selection history. The NL line has consistently been selected for both production and reproduction traits, and since 1992 litter size has been included in the breeding goal with considerable weight. The Norwegian Duroc (ND) sire line was established in 1986 and litter size has only recently been included in the breeding goal, as production and meat quality traits were most important in this line. Differences in reproduction traits are observed between purebred pigs from these genetic nucleus lines. The ND sire line has a smaller litter size with on average 9.2 total number of piglets born (TNB) compared to 13.8 TNB in the NL dam line (Ingris, 2019). An increase in TNB is genetically correlated to a higher ovulation rate and a lower corpus luteum (CL) weight (Da Silva et al., 2018). In addition, it has been shown that CLs with a smaller diameter and lower weight originate from follicles having a smaller size at ovulation (Wientjes et al., 2012). This suggests that a higher TNB in NL might be correlated to smaller follicles on the ovary. Possible differences in ovarian characteristics between the breeds could additionally lead to differences in in vitro oocyte maturation (IVM) as the use of oocytes derived from larger follicles results in better in vitro embryo production (IVP) outcomes (Bagg et al., 2007; Marchal et al., 2002; Qian et al., 2001).

In the past decades, numerous studies have been conducted to improve porcine IVP (Brüssow et al., 2000; Dang‐Nguyen et al., 2011; Grupen, 2014). Supplementation of different components to maturation media has improved oocyte development to MII stage, and cleavage and blastocyst development after fertilization (Abeydeera, 2002; Hunter, 2000). Completion of both nuclear and cytoplasmic changes during oocyte maturation is essential to acquire oocyte developmental competence (Eppig, 1996) and while nuclear maturation can be analysed with nuclear staining, cytoplasmic maturation requires an indirect assessment. Among other parameters, migration of cortical granules (CGs) is often analysed to assess cytoplasmic maturation, whereby a distribution of CGs in the cortical cytoplasm is specific for immature oocytes while a distribution just beneath the plasma membrane is specific for mature oocytes (Cran & Cheng, 1985; Pawlak et al., 2012; Wang et al., 1997). CG exocytosis, after sperm penetration of the oocyte, is important to prevent polyspermy during fertilization, and migration of CGs during maturation is therefore required. In addition, intracellular glutathione (GSH) content of oocytes, which reaches a peak level at MII stage, has been used as an indicator for cytoplasmic maturation since a higher GSH level is positively associated with male pronucleus formation (Yoshida et al., 1993), blastocyst development and total blastocyst cell number (Abeydeera et al., 1998; Maedomari et al., 2007). Moreover, cumulus cells and cumulus expansion play an important role in oocyte maturation as reviewed by Tanghe et al. (2002) and a greater cumulus expansion has been correlated with a higher oocyte developmental potential in different species (Han et al., 2006; Qian et al., 2003).

A study in other pig breeds reported a significant lower number of preovulatory follicles in Hungarian Mangalica gilts, which have a relatively poor reproductive ability, compared to Landrace gilts (Rátky et al., 2005). Mangalica oocytes showed in addition a lower degree of cumulus expansion and fewer were matured, which suggested that reproductive ability was affected by a decreased follicular development and a prolonged intrafollicular oocyte maturation. To our knowledge, in vitro maturation potential of oocytes from the ND and NL breeds with different in vivo fertility have not been studied before. Therefore, the aim of this study was to compare 1) ovarian characteristics, 2) nuclear oocyte maturation and 3) cytoplasmic oocyte maturation by assessing CG distribution and intracellular GSH, between the ND sire and NL dam line.

## 2 MATERIALS AND METHODS

### 2.1 Chemicals and media

All chemicals and reagents were purchased from Sigma‐Aldrich (Oslo, Norway) unless stated otherwise. Medium used for washing cumulus‐oocyte complexes (COCs) was Porcine X Medium (PXM) supplemented with 4.0 mg/ml bovine serum albumin (BSA) and maturation was performed using Porcine Oocyte Medium (POM) as described by Yoshioka et al. (2008) with minor modifications. The POM medium was composed of 108 mM NaCl, 10 mM KCl, 0.35 mM KH_2_PO_4_, 0.4 mM MgSO_4_.7H_2_O, 25 mM NaHCO_3_, 5.0 mM glucose, 0.91 mM Na‐pyruvate, 2.0 mM Ca‐(lactate)_2_ · 5H_2_O, 2.0 mM L‐glutamine, 5.0 mM hypotaurine, 20 ml/l BME amino acids, 10.0 ml/l MEM non‐essential amino acid, 0.6 mM L‐cysteine, 0.01 mg/ml gentamicin, 4.0 mg/ml BSA, serum substitute, 10 ng/ml epidermal growth factor (EGF) and 50 µM Mercaptoethanol (Gibco).

### 2.2 Animals

Ovaries were collected at a commercial abattoir from 37 Duroc and 20 Landrace sows originating from two Norsvin nucleus herds. Since material was collected from animals that were routinely slaughtered, no ethical approval was required. Animals were cared for according to internationally recognized guidelines and regulations for keeping pigs in Norway (The Animal Welfare Act, 10 July 2009 and Regulations for keeping pigs in Norway, 18 February 2003). Lactating sows in both herds were liquid fed with a commercial diet with whey supplementation and feed was offered up to four times a day to ensure ad libitum access during lactation. Data were collected in three replicates from June to October 2019.

### 2.3 Ovarian characteristics and genotyping

Follicular phase sow ovaries were collected 1 day after weaning and transported to the laboratory in 0.9% NaCl containing 2.5 µg/ml kanamycin at 30–35°C within 2 hr of slaughter. Ovaries were dissected free of non‐ovarian tissue before ovary weight and length were measured. The number of 3 to 8 mm follicles was counted per ovary during aspiration, and surface follicles smaller than 3 mm were subsequently counted. A tissue sample from each individual ovary was stored at −80°C for genotyping to identify the nucleus sows as it was not possible to track the animals along the slaughter line. In this way, ovaries could be matched to sow identity and ovarian characteristics could be analysed based on parity. DNA extraction from the ovaries was performed by BioBank, Hamar and genotyping was performed at CIGENE, Norwegian University of Life Sciences, Ås in Norway. Samples were genotyped using the Illumina GeneSeek custom 50K SNP chip (Lincoln, NE, United States).

### 2.4 Oocyte collection and in vitro maturation

Follicles with a diameter of 3 to 8 mm were aspirated with an 18‐gauge needle and 10 ml syringe. Oocytes with a compact cumulus and evenly granulated cytoplasm were selected and washed three times in PXM and twice in POM medium. Groups of 40 to 50 oocytes were transferred into each well of a Nunc four‐well dish containing 500 µl of pre‐equilibrated POM medium.

For the first 20 hr, COCs were matured in POM supplemented with 0.05 IU/ml porcine FSH and LH (Insight Biotechnology Ltd, Wembley, UK), and 0.1 mM dbcAMP. Subsequently, COCs were matured for another 28 hr in POM without hormones and dbcAMP. Oocytes were cultured for a total of 48 hr at 38.8°C under an atmosphere of 6% CO_2_ in humid air.

### 2.5 Assessment of cumulus expansion

At 0, 20 and 48 hr of maturation, images were taken from each well with a Nikon SMZ1500 stereomicroscope (Nikon, Tokyo, Japan) to analyse cumulus expansion. Individual COC area of each oocyte was analysed by ImageJ software (version 1.52a; NIH, Bethesda, USA) at 0 and 20 hr, and a cumulus expansion ratio per well was determined by dividing total COC area of each well at 20 hr by the total COC area of the well at 0 hr (Costermans et al., 2019). After 48 hr of maturation, it was not possible to assess cumulus expansion per individual oocyte as cumulus cells showed a high degree of expansion and were overlapping.

### 2.6 Nuclear and cortical granule staining

Cortical granule staining was based on methods reported by Yoshida et al. (1993) and Wang et al. (1997) with a few modifications. At 20 and 48 hr, oocytes were stripped of cumulus cells by repeated pipetting, washed twice in PBS, fixed in 4% paraformaldehyde for 30 min at room temperature and washed three times for 5 min in 0.3% BSA in PBS. Oocytes were then permeabilized in 0.1% triton X‐100 for 5 min, washed twice for 5 min in PBS and stained in 100 µg/ml Peanut agglutinin (PNA) lectin from *Arachis hypogaea* conjugated with Alexa Fluor 568 (L32458, Invitrogen, Thermos Fisher Scientific, Waltham, MA, USA) for 30 min at room temperature in the dark. After this staining step, oocytes were washed twice for 5 min in 0.3% BSA, 0.01% triton X‐100 in PBS, moved to a droplet of PBS and stained with 8 µg/ml Hoechst (H‐33342, B2261, Sigma). Oocytes were mounted in 6 µl fluorescence mounting medium (Dako, Glostrup, Denmark) on glass slides and a coverslip was placed on top. Slides were analysed using a Leica SP8 laser scanning confocal microscope (Leica Microsystems GmbH, Wetzlar, Germany). Hoechst staining was evaluated with a 405 nm excitation laser and a 410 to 480 nm emission filter and PNA lectin Alexa Fluor 568 with a 552 nm excitation laser and a 650 to 720 nm emission filter. Images of each oocyte were taken at the equatorial plane and at the top.

### 2.7 Assessment of nuclear maturation

Nuclear maturation of oocytes was assessed at 20 and 48 hr by Hoechst staining and confocal microscopy. Samples were analysed according to morphological criteria for meiotic stages; GV0 ‐GV4, MI (metaphase I), AI/TI (anaphase I/telophase I), MII (metaphase II) and D (degenerated) oocytes (Appeltant et al., 2015; Sun et al., 2004). At 48 hr, maturation rate was expressed as the number of AI/TI and MII stage oocytes divided by the total number of oocytes in culture. Oocytes arrested at the GV stage or only progressed to MI were considered as immature.

### 2.8 Assessment of cytoplasmic maturation

#### 2.8.1 Cortical granule distribution

**FIGURE 1:**
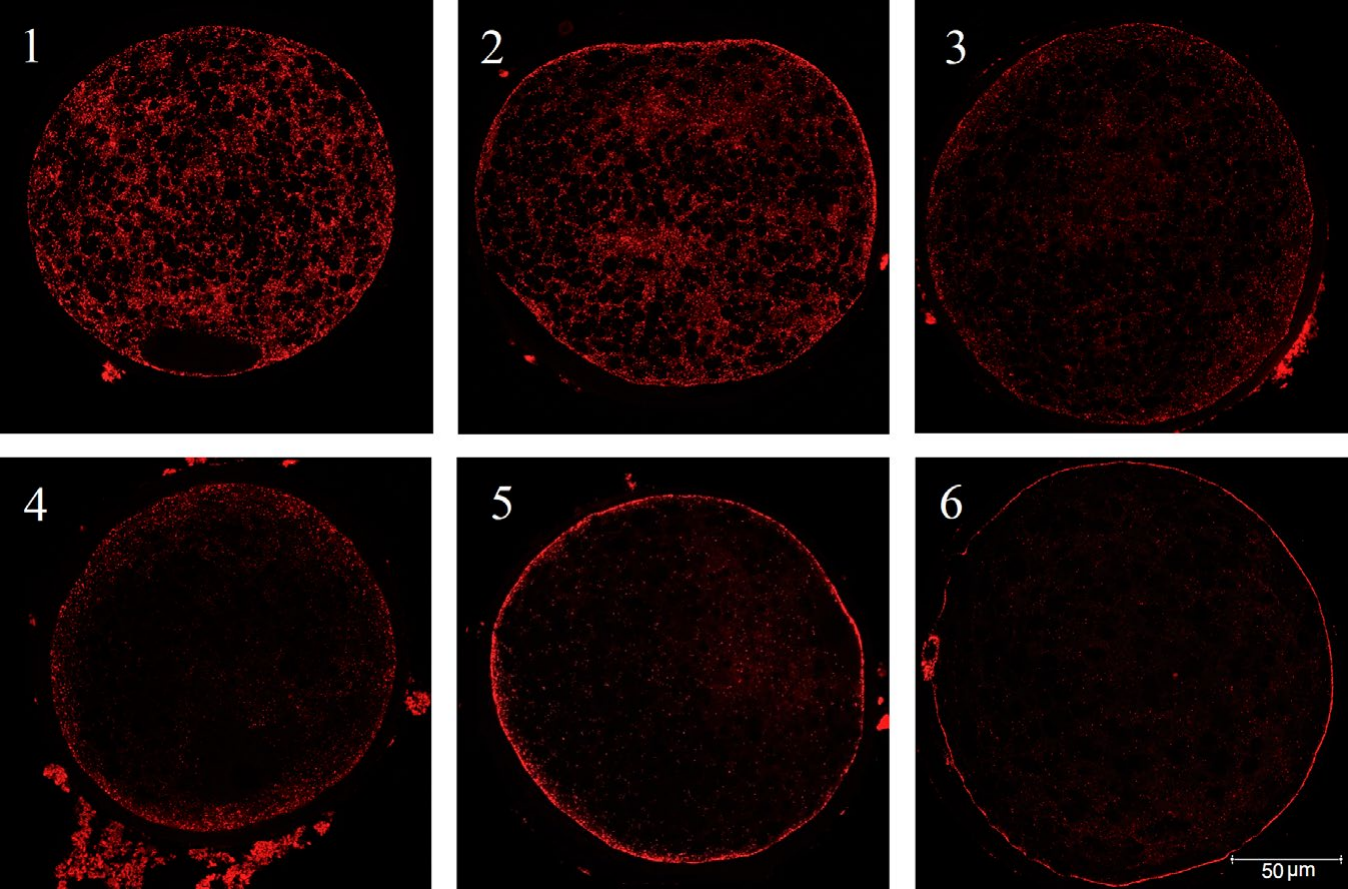
Classification of cortical granule distribution at the equatorial plane (PNA lectin). Oocytes were classified into one of the six categories; (1) Central, CGs distributed through the whole cortical cytoplasm, (2) Central distribution of CGs but already a layer under the plasma membrane in the periphery visible, (3) Outer layer in periphery and fewer CGs in the cytoplasm, (4) Clear outer layer and only a few CGs in the cytoplasm, (5) Partially complete, a thicker layer in the peripheral region, (6) Complete, a clear and thin layer under the plasma membrane in the peripheral region and around the polar body

Cortical granule distribution of oocytes was assessed at 0 and 20 hr by PNA lectin Alexa Fluor 568 staining and confocal microscopy. Classification is often based on only two or three different CG distribution patterns (Pawlak et al., 2012; Wang et al., 1997), but in this study, a distinguish was made between six different distribution categories. Patterns of CG distribution from images at the equatorial plane were classified simultaneously by two persons into one of the six categories described in Figure 1. Oocytes showing an abnormal CG distribution due to problems with mounting were not included in the CG results.

#### 2.8.2 Intracellular glutathione content

Total GSH content was determined from 28 Duroc and 26 Landrace oocytes at 48 hr of maturation. Oocytes were stripped of cumulus cells by repeated pipetting and the zona pellucida was removed using 0.05% Pronase in PBS. Oocytes were stored during every replicate in a ratio of one oocyte in 5 µl dH_2_O and frozen at −20°C. Samples were frozen and thawed three times for lysis of the oocytes to get a homogeneous mixture. Analysis was performed using the GSH/GSSG‐Glo™ Assay (Promega, Madison, WI, USA) according to the manufacturer's manual. Luminescence was measured using a Fluostar Optima multiwell plate reader with Optima control software, version 2.20 (BMG LabTech, Ortenberg, Germany). The value for each sample, measured in relative luminescence units (RLU), was converted to the corresponding GSH value in pmol per oocyte using the standard curve values.

### 2.9 Statistical analysis

Only data from first parity sows (*n* = 11 ND and *n* = 10 NL sows) were used for analysis of ovarian characteristics as different parities were not equally represented across breeds and it was therefore not possible to include parity in a statistical model. Data on IVM were based on oocytes derived from ovaries from all sows (*n* = 37 ND and *n* = 20 NL sows). All data were analysed using SAS 9.4 (SAS Inst. Inc., Cary, NC, United States). Distributions of means and residuals were examined to verify model assumptions of normality and homogeneity of variance. Data for individual COC area of each oocyte at both time points were log transformed to obtain normality before statistical analysis. Mean values for ovarian characteristics, individual COC area, COC expansion ratio and GSH content between the breeds were analysed using the Student's *t*‐test for two independent samples. Proportion of oocytes in the different nuclear maturation stages and distribution classes of CGs were analysed between the breeds using Pearson's Chi‐square test. Results are presented as mean ± *SD* and a probability of *p* < 0.05 was considered to indicate statistical significance.

## 3 RESULTS

### 3.1 Ovarian characteristics

In total, 32 Duroc and 14 Landrace sows could be identified by genotyping with an average parity of 1.9 ± 1.3 (ranging from 1 to 6) and 1.1 ± 0.3 (ranging from 1 to 2), respectively. Data from first parity sows only were used for analysis of ovarian characteristics. A larger average ovary length was observed in first parity Landrace sows compared to Duroc (*p* = 0.01), whereas no significant difference was observed in ovary weight between the two breeds (Table 1). Furthermore, on average eight additional 3 to 8 mm sized follicles were found on the surface of Landrace ovaries compared to Duroc ovaries (*p* < 0.001).

**TABLE 1:** Ovarian characteristics from 11 Norwegian Duroc (ND) and 10 Norwegian Landrace (NL) first parity sows

Characteristics	Breed	Ovaries	Mean ± SD	Min	Max	*P*‐value
Length (cm)	ND	22	3.0 ± 0.3	2.2	3.6	0.01
	NL	20	3.2 ± 0.3	2.8	3.6	
Weight (gr)	ND	22	4.5 ± 1.0	2.7	6.8	0.24
	NL	20	4.8 ± 1.0	3.3	6.6	
Number of < 3 mm follicles^1^	ND	22	12.3 ± 8.4	0.0	37.0	0.13
	NL	20	15.9 ± 5.9	8.0	28.0	
Number of 3 to 8 mm follicles^2^	ND	22	13.6 ± 5.4	5.0	24.0	<0.001
	NL	20	21.6 ± 7.9	9.0	38.0	

^1^ Total of 271 ND and 317 NL follicles counted

^2^ Total of 300 ND and 431 NL follicles counted

### 3.2 Cumulus expansion

**FIGURE 2:**
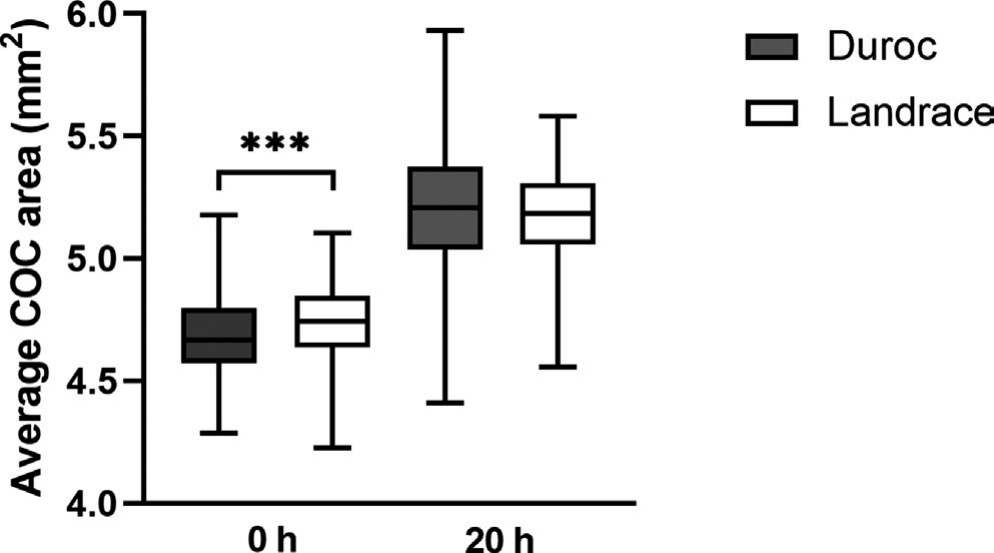
Average cumulus‐oocyte complex (COC) area at 0 hr and 20 hr of maturation for 375 Norwegian Duroc and 304 Norwegian Landrace COCs. The data were log transformed before analysis. ***p < 0.0001

**FIGURE 3:**
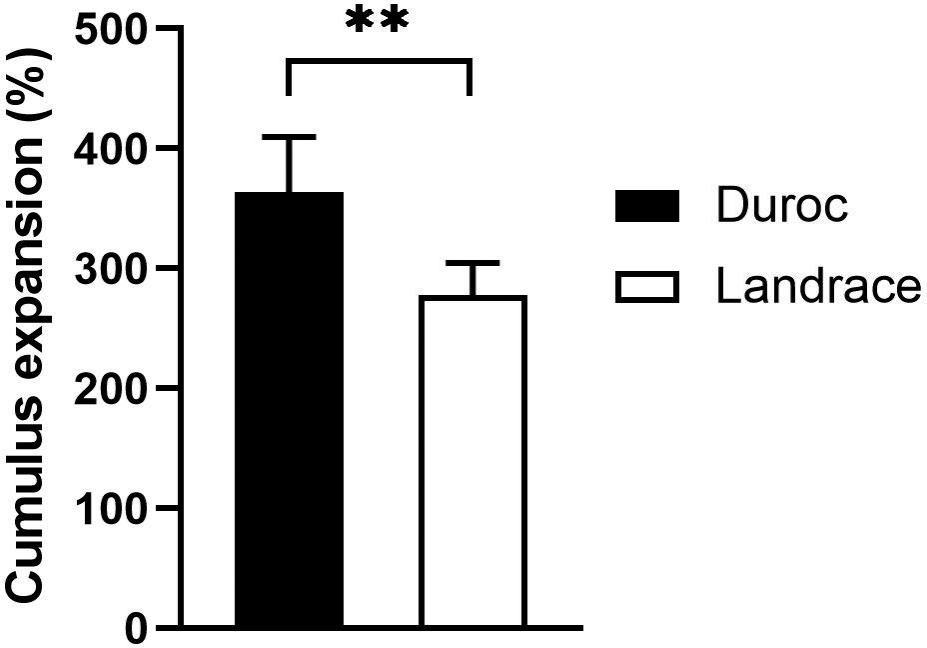
Cumulus expansion ratio (%) from 0 hr to 20 hr of maturation for Norwegian Duroc and Norwegian Landrace cumulus‐oocyte complexes (COCs). The cumulus expansion ratio was defined as the total COC area per well at 20 hr divided by the total COC area per well at 0 hr. **p < 0.001

Cumulus expansion was analysed for 375 ND and 304 NL oocytes. Average individual COC area per oocyte was significantly smaller for Duroc compared to Landrace at 0 hr (*p* < 0.0001). Contrary, average individual COC area tended to be larger for Duroc at 20 hr compared to Landrace COC area (*p* = 0.06). A larger variation was observed for Duroc COC area at this time point (Figure 2). A significantly higher cumulus expansion ratio was observed after 20 hr of maturation for Duroc COCs compared to Landrace COCs, 364 ± 46% and 278 ± 27%, respectively (Figure 3).

### 3.3 Nuclear maturation

**FIGURE 4:**
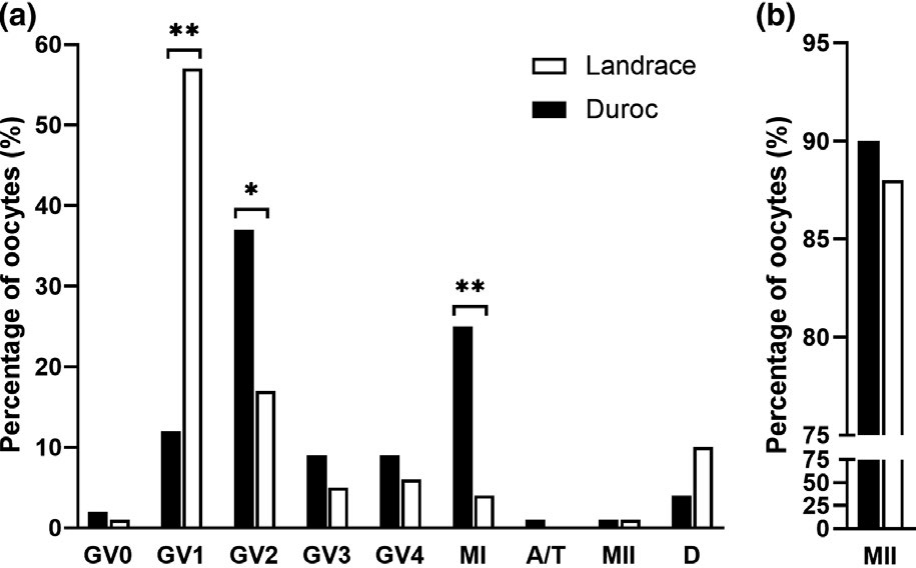
Nuclear morphology of Norwegian Duroc (ND) and Norwegian Landrace (NL) oocytes. Chromatin configuration of oocytes was classified in the categories; germinal vesicle stage 0 (GV0), 1 (GV1), 2 (GV2), 3 (GV3), 4 (GV4), metaphase I (MI), anaphase/telophase (A/T), metaphase II (MII) or degenerated (D). A) Oocytes at 20 hr of maturation (114 ND and 109 NL oocytes). B) Maturation rate to MII at 48 hr (151 ND and 162 NL oocytes). * *p* < 0.01; *** *p* < 0.0001

At 20 hr of maturation, Duroc oocytes exhibited advanced stages of maturation based on chromatin configuration compared to Landrace oocytes. A significantly higher percentage of Duroc oocytes was found in the GV2 and MI phases at 20 hr compared to Landrace oocytes, while more Landrace oocytes were present in the GV1 stage (Figure 4a). Maturation rate to MII stage, recorded at 48 hr, was not significantly different between the breeds. The MII rate for both Duroc and Landrace oocytes was consistently high, 90.1% (136/151) and 87.7% (142/162), respectively (Figure 4b).

### 3.4 Cytoplasmic maturation

At 20 hr of maturation, significantly more Landrace oocytes were classified to the CG distribution group 4 compared to Duroc oocytes, while more Duroc oocytes were found in group 3 (Table 2). In addition, analysing the proportion of all distribution groups ≥4 showed more Landrace oocytes in the more advanced distribution groups compared to the Duroc oocytes (*p* = 0.0016; ND = 34% and NL = 56%), suggesting that Landrace oocytes showed advanced stages of CG distribution at 20 hr. No significant difference between the breeds for the distributions was observed at the end of maturation.

**TABLE 2:** Percentages of oocytes derived from 37 Norwegian Duroc (ND) and 20 Norwegian Landrace (NL) sows classified in six different cortical granule distributions at 20 hr and 48 hr of maturation

Distribution	20 hr^1^ (%)		48 hr^2^ (%)	
	ND	NL	ND	NL
1	10	11	0	0
2	25	24	5	6
3	31^a^	9^b^	0	0
4	11^a^	21^b^	1	3
5	23	35	1	2
6	0	0	93	89

Values per time point in the same row with different superscript letters represent a significant difference (*p* < 0.05).

^1^ Total of 112 ND and 106 NL oocytes analysed

^2^ Total of 147 ND and 161 NL oocytes analysed

**FIGURE 5:**
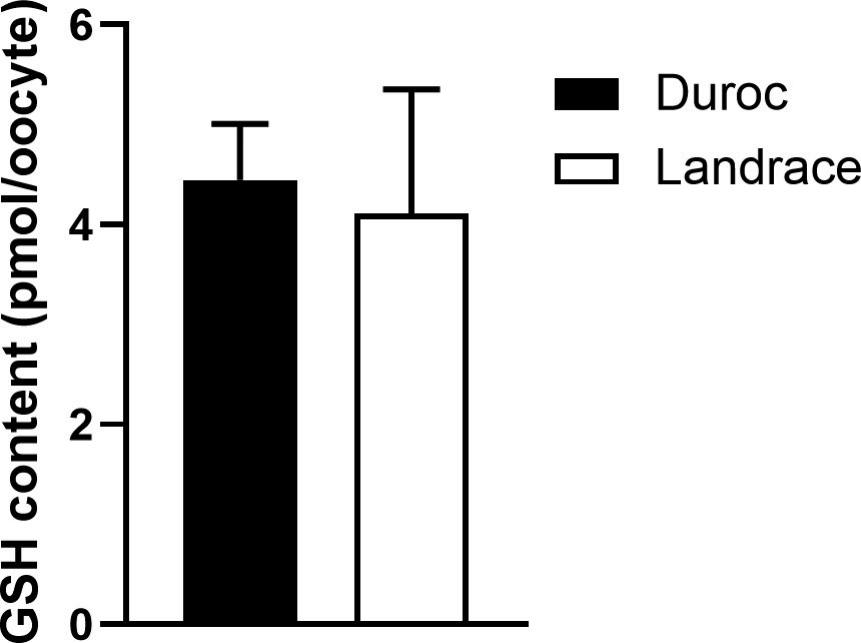
Average total glutathione content (GSH) for 28 Norwegian Duroc and 26 Norwegian Landrace oocytes at 48 hr of maturation

At the end of maturation, total GSH concentration was assessed to further elucidate cytoplasmic maturation. Total GSH at 48 hr was on average 4.45 ± 0.56 pmol per Duroc oocyte and 4.11 ± 1.25 pmol per Landrace oocyte (Figure 5). No significant difference was observed between the breeds.

## 4 DISCUSSION

This study evaluated differences in ovarian characteristics, and IVM between the ND sire and NL dam line. First parity Landrace sows had on average eight surface follicles (3–8 mm) more per ovary compared to Duroc. At a phenotypic level, this observation can explain the difference in total number of piglets born and is in agreement with selection in the breeding program, since litter size has been an important trait in the breeding goal of the dam line (NL). However, with the on‐farm management system, oocytes were collected 1 day after weaning and are representative of the early follicular phase follicle population. Collecting ovaries at day 4 or 5 post weaning would therefore represent the final selected ovulatory population and provide better insights regarding litter size. Sows from both breeds were in the same phase of the oestrous cycle and the weaning‐to‐service interval, defined as the days from weaning until the first service, is similar across both breeds (5.7 ± 1.8 days and 5.7 ± 1.3 days for ND and NL, respectively; unpublished data). This suggests that follicular development and time of ovulation relative to oestrous length are similar in Duroc and Landrace animals and that the stage of follicle development at a fixed time point after weaning would be comparable between the breeds. However, results indicated furthermore that ovary length was larger for Landrace, while no difference was observed in ovary weight. Taking into account that more follicles were present on Landrace ovaries but that weight was similar, variation in follicle size within the 3–8 mm class and follicle development might therefore exist between the breeds.

Besides assessment of ovarian characteristics, IVM was studied and cumulus expansion was analysed. Results indicate that average COC area after aspiration at 0 hr was significantly smaller for Duroc oocytes compared to Landrace. However, when cultured for 20 hr and re‐evaluated, the same COCs exhibited a larger cumulus expansion than Landrace. This greater cumulus expansion ratio from 0 to 20 hr for Duroc is of interest since a broader cumulus expansion has been associated with lower polyspermy rates (Costermans et al., 2019) and higher in vitro fertilization (IVF) and embryo development rates (Marchal et al., 2002; Qian et al., 2001).

Our results suggest furthermore that, similar to cumulus expansion, nuclear maturation at 20 hr was more advanced for Duroc oocytes compared to Landrace. At this time point, Landrace oocytes were found in earlier stages of nuclear maturation while having a smaller cumulus expansion, which might be related to a smaller follicle size at collection. A large variation in nuclear morphology at aspiration has been observed in porcine oocytes (Funahashi et al., 1997) and it would have been of interest to determine if variation in nuclear stage between the breeds already existed at the start of IVM. Nuclear maturation to MII stage at 48 hr was high in both breeds with 90.1% for Duroc and 87.7% for Landrace, and no difference was observed anymore between the breeds. This is not in line with observations from Rátky et al. (2005), who observed a significant lower percentage of matured oocytes with a lower degree of cumulus expansion in the breed with a smaller litter size. However, those oocytes were matured in vivo and collected by endoscopic ovum pick up which could differ from in vitro maturation. Cumulus expansion is essential for meiotic progression (Suzuki & Saito, 2006) and it has been suggested that nuclear maturation is preceded by cumulus expansion (Torner et al., 2004). It could be that Duroc oocytes reached MII stage earlier and that more Landrace oocytes completed nuclear maturation in the last part of maturation, as porcine oocytes normally reach MII stage around 36 hr and percentages do not change any more towards the end of in vitro maturation (Kikuchi et al., 1999; Somfai et al., 2005).

Furthermore, cytoplasmic maturation was analysed by assessing CG distributions and GSH content. In contrast to nuclear maturation, more Landrace oocytes showed advanced stages of CG distribution at 20 hr compared to Duroc. No differences in CG distribution were observed after maturation and almost all oocytes showed a clear and thin layer of CG under the plasma membrane, which is important for CG exocytosis after sperm penetration to prevent polyspermy. In line with the other parameters studied, no difference in GSH content between breeds was observed after maturation. Due to a limited number of ovaries and oocytes available, GSH levels were only analysed at 48 hr. Since Duroc COCs had a larger cumulus expansion at 20 hr, a higher GSH level would be expected as cumulus cells play an important role in GSH synthesis (Maedomari et al., 2007) and as a greater expansion is positively correlated with a higher intracellular GSH content in oocytes (Furnus et al., 1998).

The results in this study suggest that it is more efficient to use Landrace ovaries for IVP as more oocytes can be collected per animal, but it is important to examine if differences at 20 hr of maturation lead to differences in in vitro fertilization (e.g. polyspermy and fertilization rates) and embryo development. Moreover, it is worth investigating if IVF should be carried out earlier for Duroc as an increased frequency of chromosomal abnormalities has been reported when oocytes were matured over a longer time period than necessary (Sosnowski et al., 2003). The larger variation observed for parity, and thus age, in Duroc sows after genotyping could have resulted in greater variation in oocyte maturation. The use of ovaries from primi‐ and multiparous sows instead of from prepubertal gilts lead to better IVP outcomes (Bagg et al., 2007; Grupen et al., 2003; Pawlak et al., 2015), and differences might also be observed with higher parity animals as they age (Krisher, 2019). Furthermore, it is relevant to assess distribution of follicle size in a further study to better understand differences between the breeds. It is of interest if a lower TNB in Duroc is related to less follicles found on the ovary surface, to further challenges during fertilization and embryo development or if there are other factors that must be considered. Brüssow et al. (2006) indicated that the presence of COCs in the oviduct influences sperm release from the oviductal sperm reservoir. Significantly more spermatozoa were found in the ampulla and isthmus when COCs were present in the oviduct compared to oviducts without COCs. Differences in the number of COCs at ovulation in Duroc compared to Landrace, as can be indicated from this study regarding the number of follicles on the ovaries, might thus affect sperm release and fertilization. In addition, studies on uterine horn and oviduct lengths could provide a better insight into the reproductive performance in both breeds.

In conclusion, differences with regard to ovarian characteristics as well as to cumulus expansion, and nuclear and cytoplasmic oocyte maturation at 20 hr were observed between the breeds. This could subsequently affect IVP outcomes even though the two breeds showed similar maturation results at 48 hr. Therefore, further experiments are required to study if there are differences in in vitro fertilization and embryo development between the ND sire and NL dam line.

## CONFLICT OF INTEREST

The authors have no conflict of interest to declare.

## AUTHOR CONTRIBUTION

**Reina Jochems:** Conceptualization; Formal analysis; Investigation; Visualization; Writing‐original draft. **Ann Helen Gaustad:** Conceptualization; Data curation; Formal analysis; Writing‐review & editing. **Louisa J Zak:** Conceptualization; Writing‐review & editing. **Eli Grindflek:** Conceptualization; Writing‐review & editing. **Teklu Tewoldebrhan Zeremichael:** Investigation; Writing‐review & editing. **Irma C Oskam:** Conceptualization; Writing‐review & editing. **Frøydis Deinboll Myromslien:** Conceptualization; Writing‐review & editing. **Elisabeth Kommisrud:** Conceptualization; Writing‐review & editing. **Anette Kristine Krogenæs:** Conceptualization; Writing‐review & editing.

## AUTHOR CONTRIBUTIONS

RJ and AHG took part in conception and design, acquisition of data and analysis and interpretation of data. LJZ, EG, ICO, FDM, EK and AKK took part in conception and design, and interpretation of data. TTZ took part in analysis and interpretation of data. RJ drafted the manuscript which was critically reviewed and approved by all authors.

## ETHICAL STATEMENT

The authors confirm that the ethical policies of the journal, as noted on the journal's author guidelines page, have been adhered to. No ethical approval was required as the material was collected from animals that were routinely slaughtered.

## Data Availability

The data that supports the findings of this study are available from the corresponding author upon reasonable request.
